# Migration Pathways, Behavioural Thermoregulation and Overwintering Grounds of Blue Sharks in the Northwest Atlantic

**DOI:** 10.1371/journal.pone.0016854

**Published:** 2011-02-23

**Authors:** Steven E. Campana, Anna Dorey, Mark Fowler, Warren Joyce, Zeliang Wang, Dan Wright, Igor Yashayaev

**Affiliations:** Bedford Institute of Oceanography, Fisheries and Oceans Canada, Dartmouth, Nova Scotia, Canada; University of California Davis, United States of America

## Abstract

The blue shark *Prionace glauca* is the most abundant large pelagic shark in the Atlantic Ocean. Although recaptures of tagged sharks have shown that the species is highly migratory, migration pathways towards the overwintering grounds remain poorly understood. We used archival satellite pop-up tags to track 23 blue sharks over a mean period of 88 days as they departed the coastal waters of North America in the autumn. Within 1–2 days of entering the Gulf Stream (median date of 21 Oct), all sharks initiated a striking diel vertical migration, taking them from a mean nighttime depth of 74 m to a mean depth of 412 m during the day as they appeared to pursue vertically migrating squid and fish prey. Although functionally blind at depth, calculations suggest that there would be a ∼2.5-fold thermoregulatory advantage to swimming and feeding in the markedly cooler deep waters, even if there was any reduced foraging success associated with the extreme depth. Noting that the Gulf Stream current speeds are reduced at depth, we used a detailed circulation model of the North Atlantic to examine the influence of the diving behaviour on the advection experienced by the sharks. However, there was no indication that the shark diving resulted in a significant modification of their net migratory pathway. The relative abundance of deep-diving sharks, swordfish, and sperm whales in the Gulf Stream and adjacent waters suggests that it may serve as a key winter feeding ground for large pelagic predators in the North Atlantic.

## Introduction

The blue shark (*Prionace glauca*) of the family Carcharhinidae is probably the most frequently caught large shark in the world oceans [1], [2], and is certainly the most frequently caught large pelagic shark in the North Atlantic [3]. Virtually all of the North Atlantic blue shark catch is caught as undirected bycatch in the pelagic longline fishery for swordfish and tuna, where it accounts for up to 50% of the total catch weight [4], [5]. In the Canadian Atlantic, the unreported bycatch of blue sharks is estimated to be about 100 times larger than the reported catch [6]. The combination of a high unreported bycatch, a high discard rate, and a significant discard mortality rate [7] means that an accurate accounting of blue shark population abundance and mortality is difficult to obtain [8], [9]. Nevertheless, the overall abundance of the population is clearly substantial [7].

Despite the ubiquity of blue sharks in commercial catches, the movements and seasonal distribution of the species outside of fished areas is poorly understood. Catches in pelagic longline fisheries are greatest in the northwest Atlantic southwest of Newfoundland [8], [10], and southwest of Spain and northwest of Africa in the eastern Atlantic [11]; both areas appear to be preferred habitats for swordfish (*Xiphias gladius*) and occur well offshore. Blue sharks are known to be highly migratory, with tagging results suggesting that there is a single well-mixed population in the North Atlantic [12]. Trans-Atlantic migrations have been frequently documented, although most tagged sharks were recaptured either in the area they were tagged or in the central Atlantic [8], [13]. Despite the thousands of blue sharks that have been tagged in the North Atlantic, the observed recapture locations are known to be a biased indicator of movement; since recaptures can only occur in areas that are fished, recapture locations are heavily biased towards the most heavily-fished areas. In areas that are not fished at all, recaptures are clearly impossible, even if tagged sharks are abundant at that location.

As one of the most abundant apex predators in the world oceans, the blue shark undoubtedly plays a significant role in the marine ecosystem of the North Atlantic. Therefore, an understanding of the species' seasonal movements may provide insight into the predator-prey dynamics of the North Atlantic. The overwintering distribution is of particular interest in light of the the limited, fishery-independent data available for blue sharks in the winter/spring period, a period during which fishing effort is minimal and the sharks have migrated away from the continental shelf. Therefore, the objectives of this study were to: 1) use archival satellite pop-up tags to reconstruct the migration pathways towards the overwintering grounds of blue sharks in the northwest Atlantic; 2) assess the influence of the Gulf Stream on migratory direction and efficiency; and 3) consider the physiological advantage and evolutionary value of large-scale vertical and horizontal migrations. We conclude by discussing whether the overwintering ground for blue sharks in the North Atlantic might be shared by other apex predators, and thus serve as a key feeding ground for multiple species.

## Materials and Methods

### Ethics Statement

This research was conducted in accordance with the animal care guidelines of Fisheries and Oceans Canada and the Canadian Council on Animal Care.

Blue sharks were measured and tagged by scientific staff working on board commercial Canadian pelagic longliners fishing for swordfish in the fall between 2003 and 2007. The overall condition of the shark was recorded, as was its sex, fork length and maturity, as part of a study of post-release discard mortality [7].

A random sample of 40 sharks, stratified by condition at capture, were tagged with Wildlife Computers pop-up archival transmitting tags (PATs) just prior to release. All but two of the sharks were sexually immature. Tagged sharks were on deck an average of about 3 min for tagging and measurement, and showed no obvious injury above and beyond that of capture. Model 4 PATs were deployed in 2004 to 2005, while Mk-10 PATs were deployed in 2006 to 2007. PATs were attached to blue sharks by darting a nylon umbrella tip about 8 cm into the dorsal musculature of the shark just lateral to the posterior end of the first dorsal fin. The angle of dart insertion was such that the umbrella tip engaged the pterygiophores immediately underneath the dorsal fin, thus reducing the possibility of premature release. The umbrella tip was attached to the PAT with a monofilament leader of 400-pound test, sheathed to reduce trauma to the shark near the point of insertion. Each PAT was also fitted with an emergency cutoff device provided by the manufacturer which physically released the tag if it went below 1800 m (which is the maximum nominal safe depth for tag operation).

PATs were programmed to record depth (±0.5 m), temperature (±0.1°C) and light intensity at 1 min intervals (model 4 PATs) and 10 sec intervals (Mk-10 PATs) for a period of 2 to 6 mo after release. The tag data were internally binned at 6 h intervals and the summarized data transmitted to an Argos satellite after release of the PAT from the shark. More than 92% of the tags transmitted successfully after release from the shark. All PATs were programmed to release from the shark if a constant depth was maintained for a period of 4 d, since a continued presence on the ocean floor would be indicative of death in an actively-swimming pelagic shark such as a blue shark [7].

Shark location at the time of pop-up was determined with an accuracy of <1 km through Doppler-shift calculations provided by the Argos Data Collection and Location Service. The reconstruction of the migration pathway between the time of tagging and pop-up was based on sea surface temperature and ambient light at depth measurements recorded by the PAT, analyzed with the state-space model *ukfsst* described by [14].

Depth-temperature measurements recorded by the PAT were used to construct time-depth-temperature contour plots, over which the time-weighted diving trajectories of individual sharks were overlaid. The grids underlying these plots were of uniform time and depth steps of 6 hours and 8 metres respectively, encompassing the entire duration and vertical range of the analysed records. Each depth-temperature observation was assigned to its closest grid point and if more than one observation was found within a 3-hr (time) by 4-m (depth) range of a certain grid point, those values were averaged before entering the grid. A linear interpolation method was used first to fill the missing data for each vertical profile or corresponding grid's column where at least partial measurements were already present. Then spatial gaps between vertical profiles were interpolated (linearly) with a mixture of observed and interpolated data. No extrapolations were made outside of the bounding data points, and the interpolation was only performed over gaps not exceeding 100 m vertically and three days temporally. This approach allowed us to reflect in the plots all collected data and avoid artefacts or excessive smoothing caused by more sophisticated techniques of data gridding.

Although the contour plots were based on discrete temperature-at-depth measurements made by the PAT, the time-weighted trajectories of individual blue sharks were based on binned time at depth measurements from the PAT, and thus do not represent the exact trajectory between time intervals.

Ocean observations and numerical models show clearly that the speed of ocean currents associated with the Gulf Stream decrease significantly with depth below the surface [15]. This introduces the possibility that there may be some advantage to the shark's diving behaviour by reducing or benefiting from advection by ocean currents. To investigate this possibility, an eddy-admitting model based on the ocean component OPA (Océan Parallelisé [16]) of the NEMO (Nucleus for European Modelling of the Ocean) was used. The model has nominal horizontal resolution of approximately ¼° in longitude with latitude increments chosen to provide roughly square grid cells everywhere. There are a maximum of 46 levels in the vertical with thicknesses increasing from 6 m at the surface to 200 m at a depth of 1750 m and reaching the maximum value of 250 m at the bottom. The spectral nudging approach [15], [17] is used to avoid model drift and ensure a realistic mean state and eddy variability.

The influence of diving behaviour on 23 ‘numerical sharks’ was evaluated after insertion into the circulation model. The numerical sharks were seeded in the model in pairs at the locations where the real sharks were tagged and released. A constant horizontal swimming speed was specified for each shark so that in the absence of any advection by ocean currents they would arrive at the observed pop-up location at the times indicated by the observations. The specified diving behaviour was the only difference between the two sharks in each of the paired releases. One of the sharks remained continuously at a depth of 35 m while the diving behaviour of the other was specified similar to the behaviour indicated by the PAT data. In particular, the second shark was given the following diving behaviour, identical for each of the diving sharks: i) at 0600 the sharks start their decent at a rate of 3 cm/s thus descending at a rate of about 500 m in 4 hours; ii) when the shark reaches the 14°C isotherm, it remains at that level until 1800 hr at which time it will start its ascent at 3 cm/s. The sharks therefore return to the 35 m level sometime during the next 5 hours with the exact timing depending on the local depth of the 14°C isotherm; iii) after reaching the 35 m depth, the shark remains there until 0600 hr at which time the vertical diving cycle is initiated again. Note that the sharks continue to swim horizontally towards their final locations throughout this cycle. The selection of the 35 m surface depth value and the 14°C isotherm as a lower depth were based on the PAT data.

## Results

Transmissions were received from 37 of the 40 PATs that were applied to blue sharks off the eastern coast of Canada between 2004 and 2007. A total of 19 of the 37 reporting tags reported on or near the programmed pop-up date, with all but one of the remainder reporting early. Statistical analysis was restricted to the 23 tags that were at liberty at least 40 days, so as to minimize the potential for monitoring injured sharks and to maximize the potential for detecting long-distance movements. The time at liberty for the analyzed tags ranged between 40 and 210 days, with a mean of 88 days (Table 1).

**Table 1:** Tag and release data from blue sharks with PAT tags.

Argos PTT	Deployed	FL^a^ (cm)	Sex	Lat release	Long release	Pop-up date	Pop-up date	Lat popoff	Long popoff	Days at	Km^c^
				dd^b^	dd^b^	(programmed)	(actual)	dd^b^	dd	liberty	Travelled
13701	08-Sep-05	183	M	44.41	−53.28	07-Dec-05	08-Dec-05	29.21	−42.67	91	1930
13703	09-Sep-05	178	M	44.11	−52.91	12-Dec-05	20-Oct-05	41.30	−51.76	41	326
34515	27-Sep-07	168	F	44.18	−62.92	26-Dec-07	12-Dec-07	40.50	−62.19	76	407
34517	27-Sep-07	190	F	44.07	−63.04	20-Feb-08	5-Mar-08	36.19	−52.97	160	1217
34519	27-Sep-07	172	F	44.00	−63.10	30-Dec-07	30-Dec-07	33.99	−60.03	94	1138
47808	08-Sep-05	178	F	44.41	−53.28	02-Dec-05	08-Nov-05	43.05	−54.89	61	194
56387	14-Sep-05	209	M	42.12	−59.31	17-Dec-05	24-Oct-05	40.53	−52.24	40	617
56390	10-Sep-05	156	M	44.27	−53.04	12-Nov-05	12-Nov-05	43.46	−42.59	63	844
56394	26-Aug-05	201	F	44.33	−63.42	24-Mar-06	24-Mar-06	21.19	−63.55	210	2566
56395	10-Sep-05	158	M	44.38	−53.33	22-Nov-05	22-Nov-05	40.68	−43.18	73	929
56397	06-Oct-06	142	M	43.88	−63.04	22-Nov-06	22-Nov-06	42.69	−62.43	47	141
66383	06-Oct-06	138	F	43.88	−63.04	27-Nov-06	27-Nov-06	35.45	−56.38	52	1096
66388	07-Oct-06	142	F	44.34	−62.49	22-Dec-06	22-Dec-06	38.64	−61.61	76	637
66390	07-Oct-06	167	F	44.21	−62.91	1-Jan-07	1-Jan-07	40.82	−45.46	86	1479
66391	08-Oct-06	125	M	44.27	−62.53	6-Jan-07	17-Dec-06	41.61	−42.81	70	1632
66393	09-Oct-06	151	F	43.92	−63.29	16-Jan-07	19-Nov-06	41.52	−63.20	41	267
66395	09-Oct-06	142	M	44.08	−63.28	26-Jan-07	26-Jan-07	34.51	−66.21	109	1092
66399	26-Sep-07	135	F	44.15	−62.82	19-Dec-07	19-Dec-07	39.94	−56.91	84	676
67736	26-Sep-07	130	F	44.13	−62.87	9-Jan-08	9-Jan-08	32.50	−60.96	105	1303
70159	26-Sep-07	129	F	44.09	−62.90	6-Feb-08	15-Jan-08	35.51	−65.40	111	976
70240	26-Sep-07	154	F	44.03	−62.98	23-Dec-07	23-Dec-07	40.86	−64.68	88	379
70242	26-Sep-07	160	F	43.98	−63.06	16-Jan-08	16-Jan-08	39.78	−51.04	112	1100
75374	26-Sep-07	167	F	43.96	−63.07	13-Feb-08	13-Feb-08	42.29	−59.14	140	363

a fork length.

b decimal degrees.

c a straight line measure between the tagging and pop-up location.

**Figure 1:**
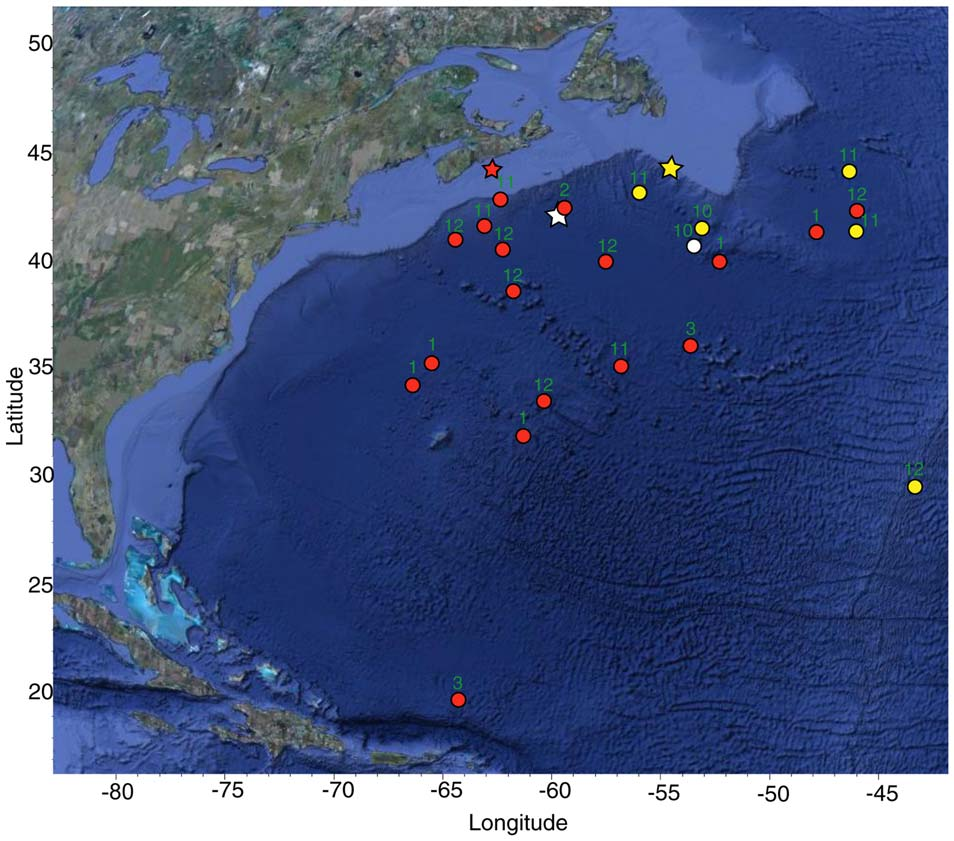
Blue shark PAT tag and pop-up locations. Map shows tagging (∗) and pop-up (•) locations for 23 blue sharks tagged off the eastern coast of Canada. Pop-up symbols are coloured to match the corresponding tagging symbol. Month of pop-up indicated by number.

All blue sharks moved off the continental shelf to the south and/or east after tagging (Fig. 1). Most sharks were north of latitude 30°N at the time of pop-up, although one travelled more than 2500 km to the southeast of Cuba (21°N). Distance travelled ranged between 141 and 2566 km (mean of 927 km), with distance travelled weakly correlated with time at large (p<0.05, r^2^ = 0.37). Mean net displacement from the tagging site was 10.8±1.2 km⋅day^−1^. There was no obvious difference in direction or magnitude of displacement between males and females.

**Figure 2:**
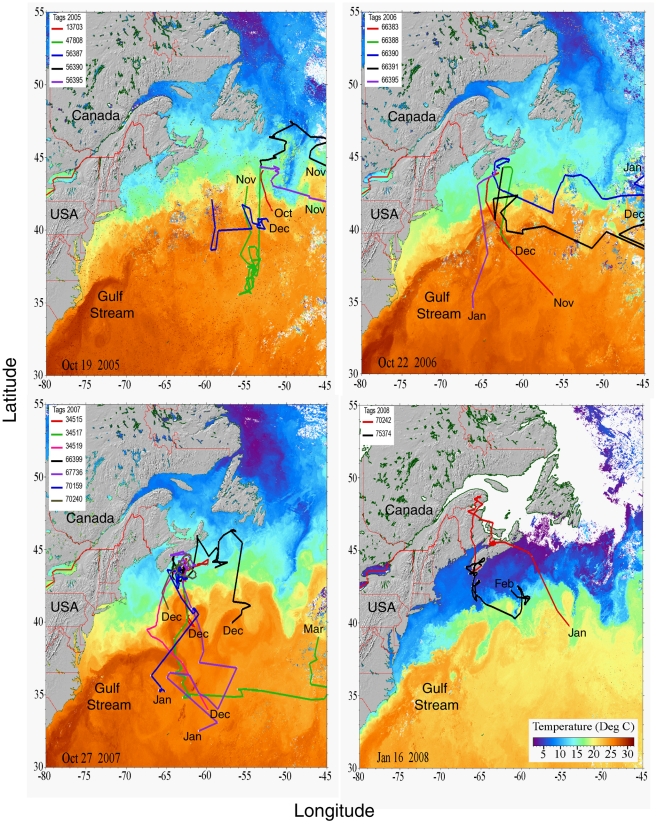
Blue shark migration pathways by year. Reconstructed migration pathways (one colour-coded solid line per shark) of blue sharks tagged with PATs, overlaid on the SST satellite imagery on the date corresponding to their presence. Sharks not entering the Gulf Stream within 2 weeks of the date corresponding to the satellite imagery are not shown. Imagery date is shown in the lower left corner of each panel, and tag pop-up month is indicated at the end of each track. Tracks of tags 56390 and 56395 (2005), 66390 and 66391 (2006), and 34517 (2007) have been truncated by 0–2 degrees at the eastern edge of the SST imagery.

Blue shark movements appeared to be closely linked to the current and temperature structure of the water. Most sharks spent the summers on or near the continental shelf, but soon encountered the warm waters of the Gulf Stream during their eastward movements (Fig. 2). In subsequent months, extending into the winter, most or all of the sharks remained in association with the warm waters of the Gulf Stream or its rings, or the Sargasso Sea further south. Since track reconstruction takes advantage of contrast in sea surface temperature (SST), movements within the relatively homogeneous temperature field of the Sargasso Sea could not be precisely estimated. Nevertheless, there were no obvious inter-year differences in migration pathways between 2005 and 2008, nor was there evidence of a single preferred pathway. However, some sharks seemed to prefer swimming in or near the front separating the warm Gulf Stream from cooler, more northerly waters.

Both the *ukfsst* track reconstruction model and examination of the depth-temperature profile received from the PAT transmission indicated that shark movements into or out of the fringes of the Gulf Stream were easily and accurately discerned. The date of entry into the Gulf Stream was estimated based on entry into water with a SST of at least 20°C, as well as a rapid increase in the SST encountered by the shark, as recorded on the PAT: a mean 5.0°C increase over one day (individual sharks experienced SST increases of 1.6–9.0°C) or 5.8°C over two days (range of 2.8–9.0°C). Based on these estimates, all but 4 of the 23 sharks entered the Gulf Stream between 14 Sept and 9 Feb, with most doing so in Oct (median date of 21 Oct). The mean dates of entry were 5 Oct (2005), 7 Nov (2006) and 12 Nov (2007), but the difference among years was not significant (ANOVA, p>0.1).

**Figure 3:**
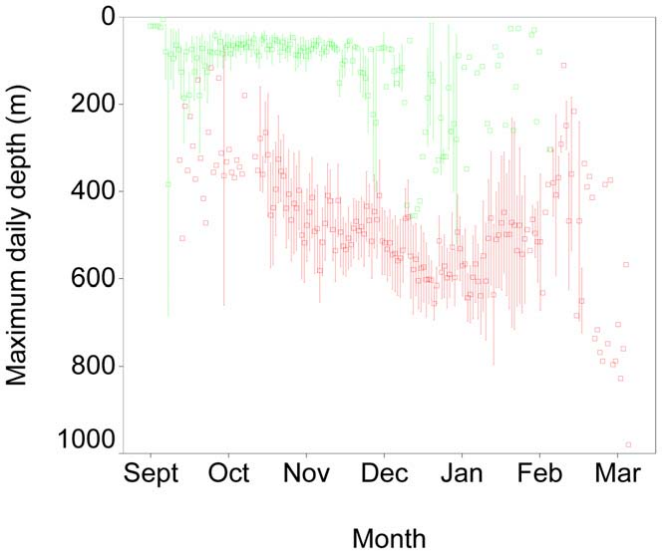
Maximum daily depth of blue sharks across months. Maximum depths varied with the month, but were much greater after entry into warm Gulf Stream waters (red) than prior to entry (green). Symbols show mean ± 1 SE.

Blue shark entry into the Gulf Stream was always accompanied by the initiation of a striking deep-diving behaviour which persisted throughout their residence in the Gulf Stream and Sargasso Sea (Fig. 3). Prior to entry into the Gulf Stream, daily maximum dive depths averaged 86±4 m (mean ± SE; n = 773), and dives exceeding 200 m were rare. However in all sharks, daily dives of more than 200 m began an average of only 1.4±1.9 days after first encountering the Gulf Stream. The initiation of deep diving behaviour was so characteristic of recent entry into the Gulf Stream that it could be used as an entry diagnostic by itself. Daily maximum dive depths while in the Gulf Stream averaged ±8 m (n = 883) with a maximum of 1008 m, and varied across months. Temperature exposure also changed after entry into the Gulf Stream, but less markedly than depth. Prior to entry into the Gulf Stream, blue sharks were exposed to a mean temperature of 15.0±0.8°C. Mean temperature increased significantly (p < 0.05) to 17.1±0.1°C after entry into the Gulf Stream.

**Figure 4:**
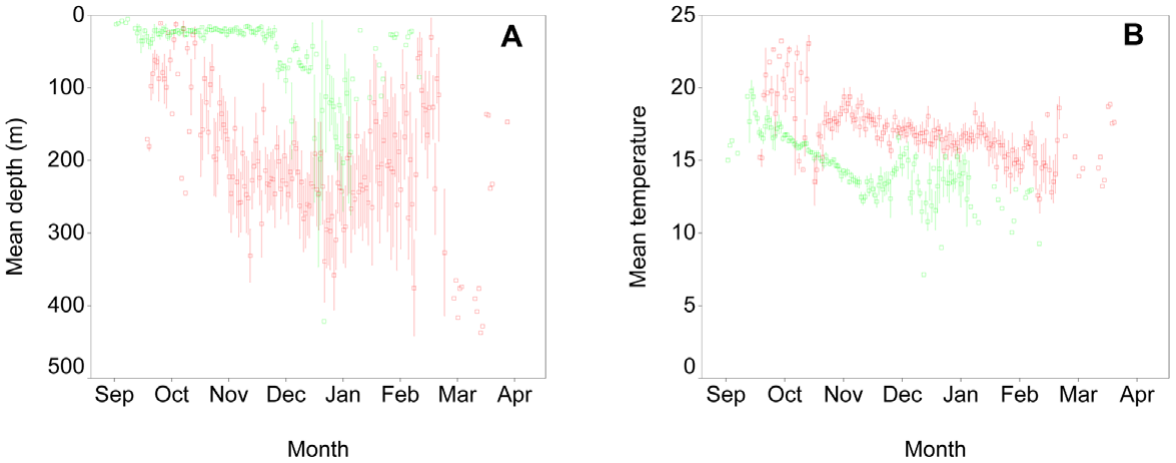
Mean depth and temperature of blue sharks across months. Symbols show mean ± 1 SE depth (A) and temperature (B) while in (red) or out of (green) warm Gulf Stream waters.

A more detailed analysis of the shark diving records revealed that the deep dives were associated with more than just proximity to the Gulf Stream, but with time of day, day of year, and the depth-temperature profile. Prior to entry into the Gulf Stream, blue sharks tended to remain in surface waters until the water temperature declined to 12–13°C in Nov, at which point most of the sharks moved into significantly deeper waters (Fig. 4). In contrast, sharks within the Gulf Stream tended to move progressively deeper between Oct and Jan. In any given month, sharks within the Gulf Stream swam in deeper and warmer waters than those outside the Gulf Stream.

**Figure 5:**
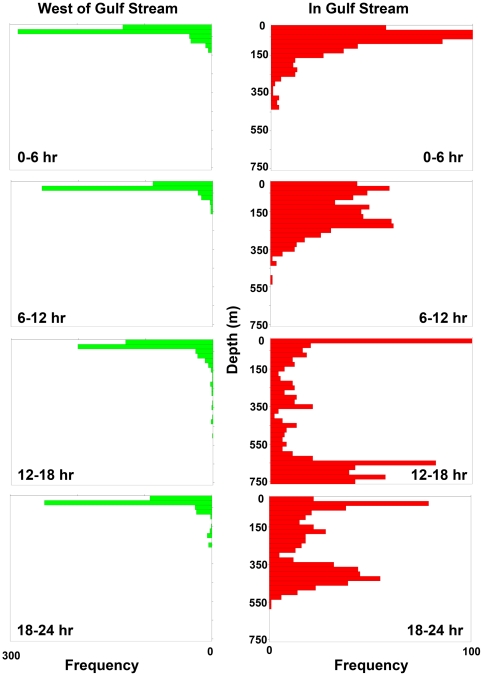
Frequency distribution of depths occupied by blue sharks by time of day. Panels show depth frequencies by 6-hr interval while in (red) or out of (green) warm Gulf Stream waters. Sharks only show extensive diel vertical migration while in the Gulf Stream, and tend to be deepest during daylight hours.

**Figure 6:**
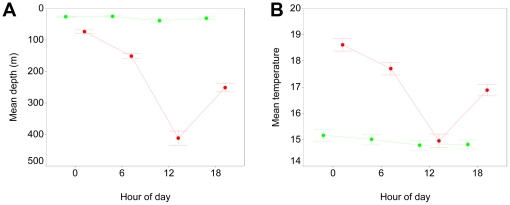
Diel changes in vertical distribution of blue sharks. Mean ±95% CI depth (A) and temperature (B) of blue sharks by 6-hr interval while in (red) or out of (green) warm Gulf Stream waters. Sharks only show extensive diel vertical migration while in Gulf Stream.

**Figure 7:**
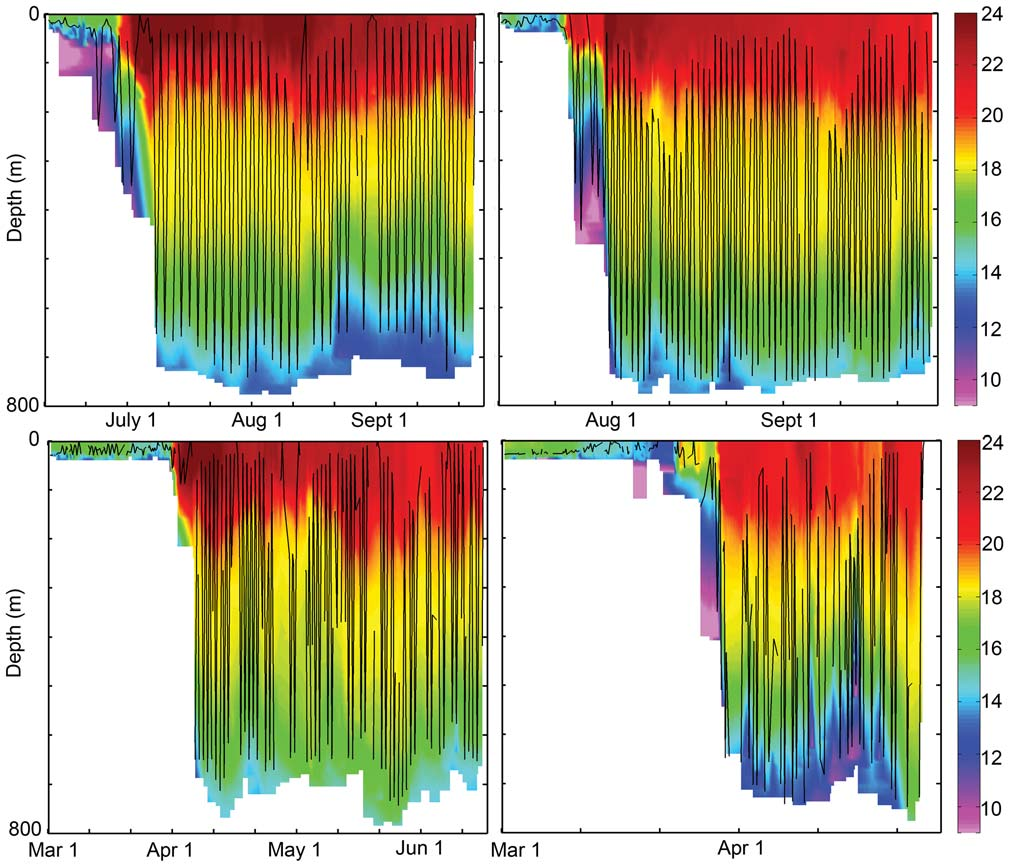
Examples of blue shark dive profiles overlaid on the temperature field. Time-weighted depths of individual blue sharks (solid black lines) from 2006 (left panels) and 2007 (right panels) at 6-hr intervals, overlaid on the colour-coded water temperature field as recorded by the PAT. Note the initiation of daily deep diving behaviour shortly after encountering the warm surface waters of the Gulf Stream.

Diel vertical migration was apparent in blue sharks at all times of the year, but was greatly amplified after entry into the Gulf Stream (Fig. 5). Outside of the Gulf Stream, there was a small but significant increase in mean depth during the daytime, from 26 m at 0000 hr to 39 m at 1200 hr, with a very small decline in ambient temperature (Fig. 6). Within the Gulf Stream however, the vertical migration was of large amplitude and exactly daily in its timing, taking the shark from a mean of 74 m at midnight, to a mean of 412 m at noon. Although there were variations in daily dive depths among and within sharks, there were few exceptions to the daily deep diving behaviour, either across days or across years (Fig. 7). Since water temperature declined with depth, the daily deep diving behaviour took sharks from surface water temperatures with a mean of 18.6°C in the nighttime, to a mean temperature of 15°C at depth (Fig. 6). Interestingly, the coolest temperatures experienced during the deep dives east of the Gulf Stream were similar to the typical temperatures experienced by the sharks west of the Gulf Stream.

Daily dive depths differed slightly but significantly with shark size, with small sharks diving to greater maximum depths (∼800 m) in the Gulf Stream than larger sharks (∼500 m) (p = 0.02, r^2^ = 0.28, n = 17). Smaller sharks also had a slightly greater time-weighted mean depth during daylight hours (1200–1800) than larger sharks (p = 0.056, r^2^ = 0.16, n = 17).

Diving depth in the Gulf Stream may have been linked to surface water temperatures, but the causal relationship was unclear. There was a significant but weak relationship between daily maximum dive depth and daily maximum surface water temperature (p<0.000, r^2^ = 0.16, n = 783), indicating that the sharks dove deeper when the surface water was warmer. However, the relationship was not apparent when the daily maximum surface water temperature exceeded 20°C, which was the temperature range most characteristic of the Gulf Stream. There was also a significant relationship between the daily maximum temperature and the daily temperature range, indicating that the sharks dove through a greater temperature range when they were warmer (p<0.000, r^2^ = 0.18, n = 775). However, there was also a significant relationship (p<0.000, r^2^ = 0.23, n = 775) between daily maximum and daily minimum temperatures, indicating that the dives were not just to a fixed temperature. In general though, blue sharks appeared to dive deeper to cooler waters when surface temperatures were warm.

**Figure 8:**
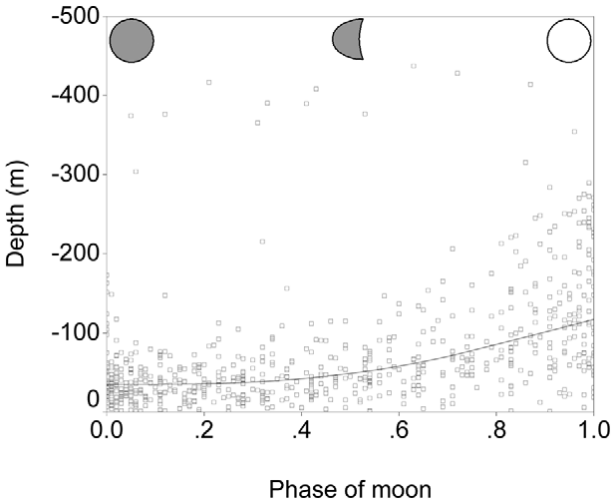
Blue shark depth at midnight versus phase of the moon. Sharks in the Gulf Stream moved to significantly greater depths as the moon became fuller. A loess curve has been fit to the data.

Blue shark depth during the nighttime tended to be shallow, but there was considerable variation between and within sharks. A significant amount of this variability in depth could be explained by illumination from the light of the moon (Fig. 8). Mean depth at midnight was three times greater during full moons compared to new moons, indicating that the sharks were deeper when the surface waters were brighter during periods of increased moon light.

**Figure 9:**
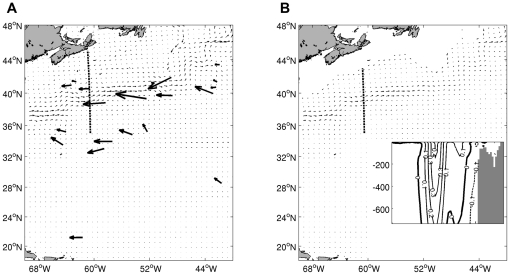
Net influence of diel diving behaviour on blue shark displacements. Ocean currents averaged over 4 years at depths of 35 m (A, thin vectors) and 600 m (B). The longest vector corresponds to a speed of 45 cm/s. Displacement vectors associated with the diving behavior are shown at the pop-up locations as bold vectors in A. Note that these displacement vectors are averaged over the full lifetime of the PATs and are normalized to give the change in displacement over a 200 day period. The inset in B shows the zonal velocities through the section indicated by the bold dashed line; solid contours indicate eastward flow while the dashed contour line indicates westward flow.

The particle drift model produced a realistic mean Gulf Stream path (averaged over 4 years) extending from its separation point of Cape Hatteras to the southern tip of the Grand Bank, with typical mean speeds of 50 cm/s and instantaneous speeds exceeding 1 m/s (Fig. 9). Along the eastern flank of the Grand Bank, the Labrador Current is squeezed between the Gulf Stream and the continental slope, carrying relatively cold fresh water southward. The inset of Figure 9 shows a typical velocity section running approximately perpendicular to the stream. After averaging, the stream width is about 200 km although its instantaneous width is typically less than half that value. Eddy variability is ubiquitous in the area of the Gulf Stream with root-mean-square current speed variations being similar to the mean speed.

The results of the particle drift model do not support the hypothesis that blue sharks dive deep while in the Gulf Stream to modify or enhance their migration speed or direction. The bold vectors shown in the left panel of Figure 9 show the mean displacement over 200 days due to the diving behaviour. To generate these results, for each pair of diving/non-diving sharks, we have taken the total vector displacement of the diving shark minus the displacement of the non-diving shark, divided by the elapsed time and multiplied by 200 days. Thus each vector gives the result averaged over the operating time of the PAT. We note however that the displacement is due to the reduction in current speed over the diving depth of the sharks and hence varies strongly in space (Fig. 9) and over the lifetime of the PAT deployments. West of the Gulf Stream, the extent of diving is minimal so there is little effect on shark displacement. The effect increases when a shark enters the Stream from the west and begins the diel diving behaviour. South and east of the Gulf Stream, the diving behaviour continues to depths of about 600 m, but the current shear is greatly reduced or absent so the effect on displacement is again negligible.

The maximum reduction in the advective effect occurs in the core of the Gulf Stream. While near the surface, the sharks are strongly advected by a mean eastward current that reaches a maximum speed of about 50 cm/s to the east-northeast for most sections across the stream. At a depth of 500 m, the maximum current speeds in the core of the stream are typically reduced by about 40% compared to their surface values and the reduction reaches roughly 80% at 1000 m (see Fig. 9). The change in mean current speed can be about 20 cm/s over 500 m. As a rough estimate, the sharks experience this difference in speed for about one third of each day and half of this difference averaged over the ascent and descent times resulting in a net westward displacement (or reduced eastward displacement) on the order of 10 km/day while in the core of the Gulf Stream. Unfortunately, the ability to determine blue shark location while in the Gulf Stream was not sufficiently precise for the model to account for spatial variations in their displacement while in the Stream. Nevertheless, the detailed model results are generally consistent with the order of magnitude estimates discussed above. On average, over the whole lifetime of the PATs, the sharks experienced a net displacement of 300 km to the northwest over 200 days, over and above what they would have experienced if they had stayed near the surface (Fig. 9). Although substantial, it does not appear that this net change in migration trajectory would have significantly altered their migratory path compared to their surface trajectory. Presumably, a diving behaviour which kept them at depth for 24 hr per day, rather than 12 hr per day, would have been used if the diving was intended to maintain a particular location. Further, we note that the diel diving behaviour continues after the sharks exit the Stream to the east and there is no significant effect of this behaviour on the sharks displacement in this region since the current shear is negligible. Inclusion of eddy variability associated with warm and cold core rings increased the variability of the shark displacement vectors, but did not appreciably change the overall pattern.

## Discussion

Previous studies based on conventional tags [6], [13], [20], [21] suggested that blue sharks overwinter offshore, but do not necessarily cross the Atlantic. Using fishery-independent data, our results confirm the previous studies with some modifications; the overwintering grounds of immature blue sharks from the northern sector of the northwest Atlantic apparently lie in the warm waters of the Gulf Stream and in the central North Atlantic as far south as the Sargasso Sea. Almost all of the satellite-tagged sharks moved offshore to the southeast or east in the fall or early winter, where they remained for periods of up to six months. Based on U.S. tagging data, 92% of blue sharks tagged in the Northwest Atlantic were recaptured in the Northwest Atlantic, with only 4% undertaking trans-Atlantic migrations [13]. Within this region however, Kohler at al. (2002) [20] reported that most of the blue sharks were recaptured near the continental shelf or east of the Gulf of Mexico, and not in the mid-Atlantic as was observed in our study. The winter range was thought to be east of the Gulf Stream [22]. The U.S. tagging results contrast somewhat with those documented by the Spanish pelagic longline fleet, which reported substantial southward and trans-Atlantic movements of tagged sharks on both sides of the Atlantic [23], with no obvious sign of overwintering east of the Gulf Stream. The discrepancies between the various studies were almost certainly due to the reliance on fishing effort to recapture and report conventional shark tags. The spatial distribution of fishing effort in the North Atlantic varies considerably by national fleets [24]. Since conventional tags cannot be recaptured without fishing effort, perceived migratory patterns can be strongly affected by the distribution (or absence) of fishing fleets. In contrast, satellite tag pop-up locations are unaffected by fishing effort, and thus provide a more realistic assessment of migratory patterns.

The location of overwintering grounds is also likely to vary with the summer location of the sharks. Extensive conventional tagging data off Ireland [21] indicated an almost mirror image of the northwest Atlantic movements, with the sharks moving to the west and southwest from Europe, with overwintering in the central and offshore east Atlantic. Taken together with the results from the northwest Atlantic, this would suggest that the north central Atlantic serves as a major overwintering ground for immature blue sharks from all areas of the north Atlantic, with the common feature being relatively warm waters originating from the Gulf Stream.

Our movement results are similar to those predicted by a previous migration model [25], which suggested that immature blue sharks in the northwest Atlantic would be more likely to be found at higher latitudes, and to migrate to the south or east during the fall, compared to adult blue sharks. On the other hand, migratory models predicting a clockwise movement of blue sharks around the North Atlantic (i.e. [20], [21]) were not well supported by our results, which indicated that many of the satellite-tagged sharks moved to the south and southeast (i.e. counter-clockwise), as well as to the east. Of course, our results were representative only of immature sharks over a period of less than 6 months, which may well show a different migratory pattern than mature sharks over a longer time period. Interestingly however, a clockwise migration pattern is also not evident in either the European [21] or central Atlantic [23] conventional tagging data, leaving open the possibility that the initial suggestion of clockwise movement was too strongly based on northwest Atlantic tagging, which was necessarily constrained against moving to the west due to the proximity of the coastline.

Is there support for the hypothesis that blue sharks take advantage of the major oceanic current systems to aid in their migration? Several previous studies have speculated that such might be the case [21], [22], [26], [27], and the premise seems logical. However, both the pop-up locations and our migration reconstructions indicate that any current-aided movements were limited at best. Indeed, the track reconstructions indicated that most of the sharks tended to maintain position (in terms of longitude) once the Gulf Stream had been entered or transited, rather than be swept along by the current. Clearly, some eastward movement took place, but its extent was limited compared to the prevailing currents. In addition, it is difficult to rationalize the westward movement of sharks tagged with conventional tags in the north central or northeastern Atlantic as having been aided by currents. Indeed, a significant number of westward trans-Atlantic migrations were made in periods of 2–9 months [21], [23], suggesting active countercurrent migration.

Deep diving behaviour is not limited to blue sharks, but the diving behaviour noted in blue sharks in the Gulf Stream and Sargasso Sea is unusual in several respects. Deep diving behaviour was almost exclusively associated with residency in the warm waters of the Gulf Stream and Sargasso Sea, as opposed to the equally-deep, cooler waters to the west. Despite some superficial similarities, the cause of this diving behaviour is unlike that reported for porbeagle sharks exiting the continental shelf on their way to a pupping ground in the Sargasso Sea. Porbeagles prefer much cooler waters than do blue sharks, and thus appear to dive beneath the Gulf Stream (to depths of 1360 m) to avoid warm surface waters often exceeding 20°C [28]. Blue sharks can tolerate much warmer water temperatures, and have been recorded as spending more than 10% of their time swimming in waters above 20°C [14], [15], [29, this study]. Thus it is unlikely that the warm surface waters of the Gulf Stream were avoided because they represented a physiological maximum. So why the sudden initiation of diel deep diving upon entry into the Gulf Stream, whereby each blue shark spends the night in surface waters and the days at great depths?

Although diel or periodic vertical migration has been observed in many species of shark and billfish, relatively few have reported the precisely diel vertical migrations accompanied by continued residence at depth during the daytime that were observed in this study. Blue sharks in the northeast Pacific made only brief dives below the thermocline, and none below 700 m [19]. Irregular diel diving, including multiple deep dives during the daytime to 300–500 m, have been noted in blue sharks in the southwest Pacific [18], the northwest Atlantic [30] and the northeast Atlantic [29], as well as in school sharks (*Galeorhinus galeus*; [36]), swordfish (*Xiphius gladius*) and several of the tuna species [31], [32], although none of them remained at depth for extended intervals. On the other hand, regular and persistent swimming at depth during the daytime, analogous to what was observed in the current study, has been observed in thresher sharks (*Alopias superciliosus*) [33]), some swordfish [31], and one white shark (*Carcharodon carcharias)* [34].

There are several hypotheses that could potentially explain the striking daily pattern of deep diving in blue sharks: a) foraging; b) thermoregulation; c) oxygen limitation; d) migration to take advantage of depth-related variation in current speed; e) reproduction; f) predator avoidance; and g) navigation. Of these possibilities, reproduction is unlikely to be a viable explanation, given that all but two of the tagged sharks were sexually immature. Predator avoidance is also unlikely, given that blue sharks are apex predators and thus unlikely to be predated upon. In addition, all size classes of sharks were aggregated at the same depth interval in the nighttime hours, and together at all depths in waters outside of the Gulf Stream and Sargasso Sea. Oscillatory diving behaviour has been noted in several shark species, and has sometimes been interpreted as reflecting navigation using local variations in the Earth's magnetic field [36]. However, it is hard to rationalize why the blue sharks would navigate using the magnetic field only within the confines of the Gulf Stream, even when off the continental shelf, and only during the daytime. The remaining hypotheses will be explored in more detail, beginning with those that appear least likely. In doing so, we acknowledge that no physiological data or actual behavioural observations were collected to test some of these hypotheses.

### Oxygen limitation

Oxygen minimum layers (OMLs) are found at depths of 300–1000 m in most of the world oceans. Except where the oxygen concentrations are truly depleted (<0.15 mL·L), many fishes and cephalopods are able to move into and through this depth range without specific adaptations [37]. Dissolved oxygen concentrations in the Gulf Stream around latitude 42°N reach minimum levels at depths of 200–400 m, but at concentrations exceeding 3 mL·L [I. Yashayaev, unpublished data]. Given that most of the diving blue sharks routinely swam through the OML to greater depths, it seems unlikely that the diel diving behaviour of blue sharks was somehow modified by oxygen concentrations.

### Current-assisted migration

The hypothesis that blue sharks use the major current systems in the world oceans as a migration aid [25] may hold at time scales longer than were examined in this study, but we found little evidence in support of it at time scales of less than 6 months. Even if blue sharks do use the currents to move around, the diel diving behaviour would act to reduce net migration, not enhance it. The results of our particle drift model indicated that the net effect of extended daily residency at depth served to reduce the magnitude of eastward drift with the Gulf Stream. The effect of the reduced current at depth was much less when the shark was outside the main body of the Gulf Stream. Presumably then, an adaptive strategy to take full advantage of the Gulf Stream current would keep the sharks in the surface waters where the current speeds were the greatest. Therefore, the hypothesis that the diel diving behaviour would enhance migratory capability was not supported. Although the diving behaviour clearly had a significant impact on net movement compared to continued residency in surface waters, there was little evidence that this difference was anything other than an accidental artifact of the diving behaviour.

### Foraging behaviour

There are several lines of evidence that indicate that the daily deep diving behaviour of blue sharks in the Gulf Stream is linked to foraging on vertically migrating prey, although the foraging in turn is firmly linked to thermoregulatory factors. Firstly, the daily deep diving behaviour was only associated with the warm waters of the Gulf Stream and Sargasso Sea; it was not apparent in the colder waters to the west of the Gulf Stream, even within days of entry. Therefore, any vertically migrating prey items were likely restricted only to the warmer waters. Squid are the most likely candidate here, since they are a preferred prey item of blue sharks [38]–[40]. Several species of squid are known to concentrate or spawn at depths of up to 1000 m within the Gulf Stream, and in particular, near the front with the cooler waters where the sharks were most abundant [41]–[46]. Although the vertical movements of squid in the Gulf Stream are poorly documented, studies of squid species in both the Atlantic and the Pacific indicate that diel vertical migration is the norm rather than the exception, with the squid spending the nighttime hours in the surface layers, while daytime depths are generally centred around 500–600 m, with some going to 1000 m [47]–[49]. Vertically migrating small fish prey are almost certainly present in the Gulf Stream as well, with myctophids in the central equatorial Atlantic all vertically migrating to depths of up to 1250 m during the day, with central daytime distributions centred at 400–700 m [50]. The strongly overlapping spatial distribution of overwintering blue sharks with their preferred squid and fish prey, along with vertical migration patterns which are virtually identical, provides strong evidence that the blue sharks are feeding on their vertically migrating prey near the surface at night, then diving to follow them to great depths during the day. The observation that blue shark depth under moonlight is strongly correlated with the phase of the moon is consistent with the pursuit of vertically migrating prey, which are in turn diving deeper to avoid the additional light of the full moon. Therefore, the argument that the overwintering blue sharks are spending their days at great depth to feed seems clear except for one major constraint: blue sharks appear to be functionally blind at the depths where they spend their daytime hours.

### Daytime Foraging in the Dark

The visual sensitivity of blue shark eyes has not yet been reported, but might be expected to be similar to that of another species of surface-adapted shark, the lemon shark (*Negaprion brevirostris*) [51]. Light intensity (I) at depth (z) can be calculated using Beer's Law and estimates of the light extinction coefficient (*k*) and the incident light intensity at the water surface (I_0_), where Beer's Law is:

I_z_  =  I_0_ e^−*k*z^

Assuming a *k* value of 0.033 corresponding to the clearest ocean water [54], and using published incident light values at the surface [52] or at 71 m near Bermuda [53], and given the visual detection limit of a dark-adapted lemon shark [51] as a proxy for that of blue sharks, blue sharks should be functionally blind at a depth of no more than 535–550 m Calculations based on a more realistic *k* value of 0.07 [52], [55] yields a limiting visual depth of about 255 m. Therefore, it appears likely that blue sharks become unable to see at daytime depths of between 250–550 m, implying that some or most of their daytime dives in the Gulf Stream are carried out in complete darkness (to them). A similar conclusion was reached for migrating hammerhead sharks (*Sphyrna lewini*), who appeared to be swimming and navigating at light levels too low for visual detection [56]. Since blue sharks appear to be following their vertically migrating prey down to depths of as much as 1000 m during the day, this would imply that they are either detecting their prey using non-visual senses (i.e. olfaction or electroreception) or are pursuing bioluminescent mesopelagic prey (including squid) [57] which are detectable at depths below the visual limit of the shark.

If blue sharks are following and feeding on vertically migrating prey while in the Gulf Stream, one would expect the mean depth of the sharks to closely match that of their prey. Our results for night-time depth distributions through the lunar cycle strongly supported that hypothesis, since shark depth appeared to follow the isolume: the sharks swam deeper on moonlit nights than on nights without a moon. There is no obvious reason why sharks should follow an isolume at night other than to pursue their vertically migrating prey, which are in turn following the isolume. Similar observations have been made for school shark, *Galeorhinus galeus*, [35] and swordfish [31]. An interesting corollary of this hypothesis is that the vertically migrating prey (and thus their pursuing sharks) would be expected to move deeper on bright sunny days than on cloudy days. Unfortunately, our attempt to test this hypothesis using satellite imagery of cloud cover linked to date-specific daytime shark depth was inconclusive.

Given that the deep-diving blue sharks are attempting to feed at or below the limits of light detection during the daytime, their feeding success would be expected to be somewhat less than near the surface during the night (where light levels are higher), even if (as is likely) non-visual senses are used to aid in prey detection. Below we argue that there is a thermoregulatory advantage to adopting what appears to be a sub-optimal pursuit strategy.

### Thermoregulatory Advantage of Deep-diving Behaviour

If there is a thermoregulatory advantage to the deep-diving behaviour of blue sharks, it could manifest itself as either a return to surface waters to warm up after a deep dive, or a dive to cooler deep waters to cool down. Both behaviours have been suggested previously for sharks and other large pelagic fishes, but a strict thermoregulatory explanation for deep-diving behaviour does not appear to apply directly to blue sharks. Blue sharks monitored in the central North Atlantic using telemetry revealed that the sharks were diving periodically to depths of 400 m [30]. It was suggested that the sharks were following vertically migrating prey such as octopods, and that the sharks were returning to the surface to warm up. However, the temperature at depths of 400-500 m in the Gulf Stream (∼15°C) is very similar to that occupied by blue sharks in surface waters outside of the Gulf Stream, suggesting that no warming would be required. Reverse thermoregulation, whereby the sharks dive to cool off, would appear to be more consistent with archival tag observations of bluefin tuna, who sometimes dive repeatedly through the thermocline during the day to cool off [32], [58], [59]. However, tuna anatomy includes a vascular heat exchange system which gives the tuna a thermoregulatory capability lacked by blue sharks. Nor was there any relationship between surface water temperatures above 20°C and the temperature at depth, which would be expected if overheated blue sharks needed to dive deeper to cool off to a greater extent. Finally, there was no indication that the daily dives were required because the surface temperatures were anywhere near the lethal limit for blue sharks: blue sharks in this study occupied water temperatures up to 28°C, yet deep diving behaviour was initiated at a mean surface temperature of just 20.1°C.

Although a strict thermoregulatory explanation does not seem applicable to blue sharks, behavioural thermoregulation designed to reduce metabolic losses and increase foraging efficiency appears to explain the observed blue shark behaviour. The metabolic costs of remaining in warm surface waters during the day, rather than cooler deep waters, can be estimated using the observed temperature differential between surface and deep waters in the Gulf Stream, and published values for routine metabolic rate and Q_10_ in sharks (with Q_10_ being the factor by which metabolic rate increases for every 10°C increase in temperature). The mean observed temperature differential in blue sharks diving >400 m in the Gulf Stream was 8.5°C (n = 460). Assuming a Q_10_ of 2.9 [60], and given that all blue sharks continually swim, whether or not they are changing depth, sharks remaining near the surface would expend about 2.5 times more metabolic energy than comparably-fed sharks at ≥400 metres of depth. If in fact feeding opportunities near the surface during the day are more limited than those at depth, the energy losses of sharks remaining at the surface could be even greater than a factor of 2.5. Therefore, there appears to be a strong metabolic advantage to daily vertical migration, even to depths where shark foraging efficiency is impaired by low light. Of course, predation rates, metabolic rates and body temperature measurements would need to be made before this hypothesis could be fully tested.

A foraging strategy which has blue sharks pursuing vertically-migrating squid and fish from surface waters at night to deep waters during the day, would reduce metabolic rate, increase metabolic efficiency, and thus preserve more energy obtained from food for growth, whether or not foraging at depth was limited by darkness. This hypothesis is supported by the observation that blue sharks must have been pursuing light-reacting prey when they altered their depth above the thermocline at night in response to the phase of the moon; a strictly thermoregulatory explanation would have required deep dives below the thermocline both day and night. The blue shark foraging-thermoregulatory behaviour contrasts with that reported for bluefin tuna, who appear to make numerous deep but short dives during the day to avoid overheating, although a foraging explanation could not be rejected [32], [58], [59]. Porbeagles also dive to great depths while in the Gulf Stream and Sargasso Sea [28]. However, the deep swimming depth is maintained night and day, suggesting that the cold-adapted porbeagles are avoiding warm surface waters rather than pursuing prey. A closer analogue may be chum salmon (*Oncorhynchus keta*), who appear to dive periodically to deeper, cooler waters to minimize metabolic losses (and thus minimize loss of body weight) during their non-feeding homing migration [61].

### A Winter Feeding Ground for Large Pelagic Predators?

The daily deep-diving foraging behaviour by overwintering blue sharks in the Gulf Stream and nearby waters is not normally present in other blue shark populations/habitats, suggesting that the winter Gulf Stream feeding opportunities may be better than elsewhere in the Atlantic. Squid are known to be extremely abundant in the Gulf Stream, with many species vertically migrating [47]–[49], and one of the most abundant squid taxa (*Illex* spp.) spawning in or near the Gulf Stream in the winter [45], [46]. Therefore, one might expect a suite of large predators to overwinter and dive in Gulf Stream waters to take advantage of squid and fish availability. Swordfish and bluefin tuna both engage in frequent deep dives as they migrate along the Gulf Stream during the winter [32], [62], raising the possibility that they are feeding on squid and small fish as they migrate. Sperm whales are widely distributed in the North Atlantic, but are concentrated along the north flank of the Gulf Stream [63], an area of particularly high numbers of squid. Finally, it is possible that porbeagle sharks use the Gulf Stream as a feeding ground for their newborn pups [28], which may use the Gulf Stream as a moving nursery ground for the young-of-the-year porbeagles as they are carried back to Canadian coastal waters. Since sharks, swordfish, tuna and sperm whales are the dominant large pelagic predators of the North Atlantic, and all seem to be concentrated and deep diving in the Gulf Stream and adjacent waters during the winter months, it may be that the Gulf Stream and adjacent waters is a key winter feeding ground for apex predators in the North Atlantic.
